# A circuit mechanism linking past and future learning through shifts in perception

**DOI:** 10.1126/sciadv.add3403

**Published:** 2023-03-24

**Authors:** Michael Crossley, Paul R. Benjamin, György Kemenes, Kevin Staras, Ildikó Kemenes

**Affiliations:** Sussex Neuroscience, School of Life Sciences, University of Sussex, Brighton BN1 9QG, UK.

## Abstract

Long-term memory formation is energetically costly. Neural mechanisms that guide an animal to identify fruitful associations therefore have important survival benefits. Here, we elucidate a circuit mechanism in *Lymnaea*, which enables past memory to shape new memory formation through changes in perception. Specifically, strong classical conditioning drives a positive shift in perception that facilitates the robust learning of a subsequent and otherwise ineffective weak association. Circuit dissection approaches reveal the neural control network responsible, characterized by a mutual inhibition motif. This both sets perceptual state and acts as the master controller for gating new learning. Pharmacological circuit manipulation in vivo fully substitutes for strong paradigm learning, shifting the network into a more receptive state to enable subsequent weak paradigm learning. Thus, perceptual change provides a conduit to link past and future memory storage. We propose that this mechanism alerts animals to learning-rich periods, lowering the threshold for new memory acquisition.

## Teaser

Prior learning shifts perception and enhances new memory acquisition by stabilizing activity in competitive neural circuits.

## INTRODUCTION

Learning and long-term memory (LTM) formation are important but energetically costly processes, particularly for animals on a limited metabolic budget (*1*, *2*). As such, neural strategies that could help identify the most relevant or fruitful associations are likely to be highly beneficial for survival. Neuronal mechanisms to support such guided learning, however, are not established. A candidate to help instruct new memory formation is an animal’s prior experience, but the way in which past and future learning might be linked is poorly understood. One potential insight comes from human studies indicating that prior learning tunes perception via attentional modulation, which guides perceptual processing in future tasks and enhances performance (*3*–*5*). This raises the intriguing possibility that perceptual changes could be an important conduit that serves to link past and new learning.

Simple invertebrate models have been extensively exploited to elucidate fundamental neural mechanisms for learning and action selection (*6*–*14*). Here, we used *Lymnaea*, an established molluscan system (*15*–*22*), to directly probe the relationship between past memory, perception, and new learning. This animal can rapidly form associations between stimuli after a single training session with the resulting memory lasting for several weeks (*23*, *24*). The neurons in the central nervous system are large, identifiable, and accessible, and the underlying circuits for the expression of the conditioned response have been extensively characterized (*15*, *25*–*27*), making this a compelling choice for detailed behavioral and circuit-level investigations. Here, we subjected animals to two types of appetitive classical conditioning paradigm, a strong one that induces a stable LTM and a weak one that does not. When presented sequentially (strong then weak), the conditioned stimulus used in the weak training paradigm subsequently produces a robust conditioned response. Using both behavioral and electrophysiological readouts, we demonstrate that while the stimuli used during weak training are originally perceived by the animal as ambiguous/bistable (both positive and negative), the prior strong learning shifts this perception to a stable positive state. We also identify the control microcircuit responsible, demonstrating that it works by controlling competitive interactions between two antagonistic feeding behaviors, enabling the threshold for positive learning associations to be dynamically altered. Pharmacological replication of the altered perception in vivo can fully substitute for the acquisition of the strong memory, biasing the network toward a more receptive state to enable new positive associations. Our study reveals a key mechanism for coupling past and future learning through changes in perception. We hypothesize that this serves to signal to the animal a potentially learning-rich environment, allowing new positive associations to form to cues that would otherwise be ignored.

## RESULTS

### Previous learning facilitates new memory acquisition

**Fig. 1.**
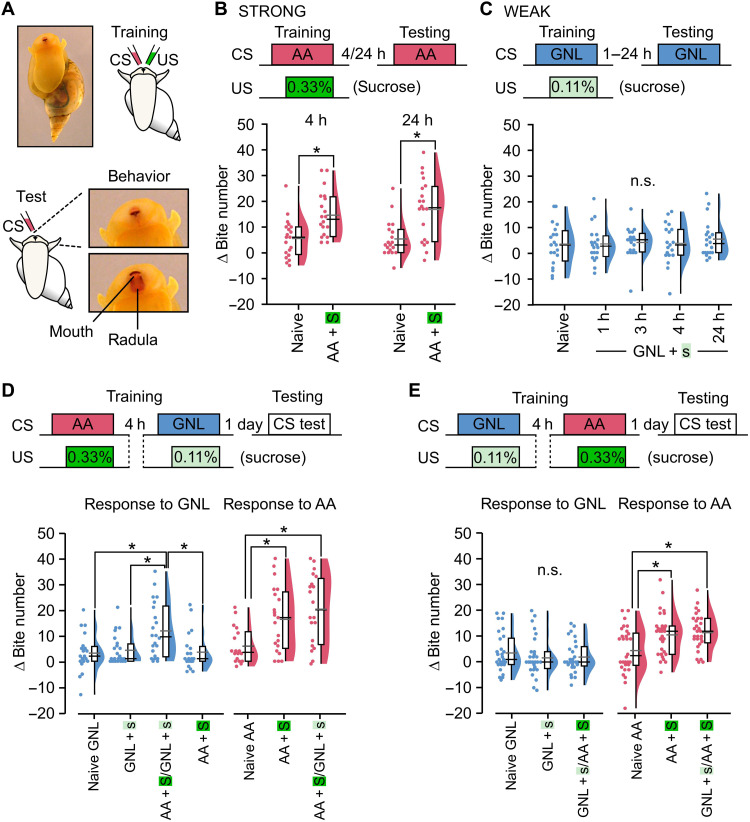
Previous strong learning enhances subsequent weak learning. (**A**) Scheme illustrating appetitive conditioning in *Lymnaea*. ∆ Bite number calculation: bites post-CS minus pre-CS. (**B**) Pairing AA (CS) with sucrose (US; 0.33%, “S”) causes a significant learned response to AA tested 4 hours (naïve; *n* = 20, 4 hours; *n* = 20, Mann-Whitney test, *P* < 0.01, *U* = 105.5) and 24 hours (naïve; *n* = 19, 24 hours; *n* = 20, Mann-Whitney test, *P* < 0.01, *U* = 81) after training. (**C**) Pairing GNL (CS) with a low-concentration sucrose (US; 0.11%, “s”) causes no significant learned response tested 1, 3, 4, or 24 hours after training (naïve; *n* = 20, 1 hours; *n* = 20, 3 hours; *n* = 20, 4 hours; *n* = 19, 24 hours; *n* = 20, Kruskal-Wallis test, *P* > 0.05, *H* = 1.2). (**D**) Strong training 4 hours before weak training causes a significant learned response to GNL versus all other conditions (naïve, *n* = 20; weak only, *n* = 24; strong 4 hours before weak, *n* = 22; strong only, *n* = 20; Kruskal-Wallis test, *P* < 0.01, *H* = 12.4; Dunn’s test: strong 4 hours before weak versus naïve, *P* < 0.01; weak only, *P* < 0.01; strong only, *P* < 0.01; all other conditions, *P* > 0.05). The response to AA was significantly increased versus naïve, but not strong training only (naïve, *n* = 18; strong only, *n* = 21; strong 4 hours before weak, *n* = 20; Kruskal-Wallis test, *P* < 0.01, *H* = 10.3; Dunn’s test: strong 4 hours before weak versus naïve, *P* < 0.01; strong only versus naïve, *P* < 0.05; strong 4 hours before weak versus strong only, *P* > 0.05). (**E**) Weak training 4 hours before strong training causes no significant learned response to GNL versus all other conditions (naïve, *n* = 30; weak 4 hours before strong, *n* = 29; weak only, *n* = 29; Kruskal-Wallis test, *P* > 0.05, *H* = 0.7). The AA response was increased versus naïve, but not strong training only (naïve, *n* = 30; weak 4 hours before strong, *n* = 29; strong only, *n* = 30; Kruskal-Wallis test, *P* < 0.01, *H* = 11.2; Dunn’s test: weak 4 hours before strong versus naïve, *P* < 0.01; strong only versus naïve, *P* < 0.01; weak 4 hours before strong versus strong only, *P* > 0.05). h, hours.

*Lymnaea* form robust and long-lasting memories after only a single pairing of a neutral and rewarding stimulus (*23*, *24*). Here, we exploited this system to examine how different learning events interact. We established two different training protocols: The first used a single pairing of amyl acetate [AA; the conditioned stimulus (CS)] and 0.33% sucrose [S; the unconditioned stimulus (US)], inducing an LTM (*17*) that was expressed as an increased feeding response to the CS tested 4 or 24 hours after training (Fig. 1, A and B; fig. S1A; and movie S1). On the basis of the robustness of the learned outcome, we classified this as a “strong” training paradigm. In the second protocol, we paired an alternative CS, gamma-nonalactone (GNL), which did not differ from AA in its effect as a neutral stimulus (fig. S1B), with a lower concentration of sucrose (s; 0.11%, US). This pairing was insufficient to induce a memory when tested at 1, 3, 4, or 24 hours after training (Fig. 1C and fig. S1C) and therefore was classified as a “weak” training paradigm. Next, we examined whether interactions between these training protocols might influence the outcome of learning. To do this, we established a double training paradigm in which animals received the strong training followed, after a 4-hour interval, by the weak training (Fig. 1D). Notably, using this protocol, we found that a robust LTM was now formed because of the weak training (Fig. 1D and fig. S2A). This outcome was not explained by stimulus generalization since strong training (AA + S) alone did not change the GNL response compared to naïve animals (Fig. 1D and fig. S2A). Moreover, memory after strong training (AA + S) was not disrupted by the weak training (GNL + s), confirming that no retroactive interference between the memories occurred (Fig. 1D and fig. S2A). The effect was also not dependent on which CS was used in the strong or weak training; reversing the paradigm to use GNL as the CS in strong training and AA as the CS in weak training also produced the same outcome (fig. S2B). In a test of the persistence of weak training memory following strong training, we demonstrated that the memory trace was still present 48 hours later (fig. S2C). Presentation of the strong US alone 4 hours before the weak training did not increase the GNL response (fig. S2D), suggesting that the effect was specifically dependent on the acquisition of the previous strong memory. To further test the necessity of prior strong memory acquisition, we performed a double training paradigm in which animals received two weak trainings 4 hours apart (AA + s/GNL + s or GNL + s/AA + s) and found that there was no memory for either CS (fig. S2E). Next, we considered the importance of the timing interval between strong and weak training. We found that weak training was sufficient to induce an LTM when presented between 30 min and 4 hours after strong training, suggesting that there is a critical post-learning time window in which new memory acquisition can be facilitated (fig. S3A). Last, we determined the importance of the temporal order of training, specifically whether strong training could retroactively enhance a memory trace induced by weak training. We found that weak training was insufficient to induce an LTM when it preceded strong training by 4 hours (Fig. 1E and fig. S3B), showing that the effectiveness of weak training critically depends on prior strong training. Together, these results suggest that previous acquisition and consolidation of a strong memory act to lower the threshold for new appetitive learning.

### Previous learning changes the perception of a new learning event

**Fig. 2.**
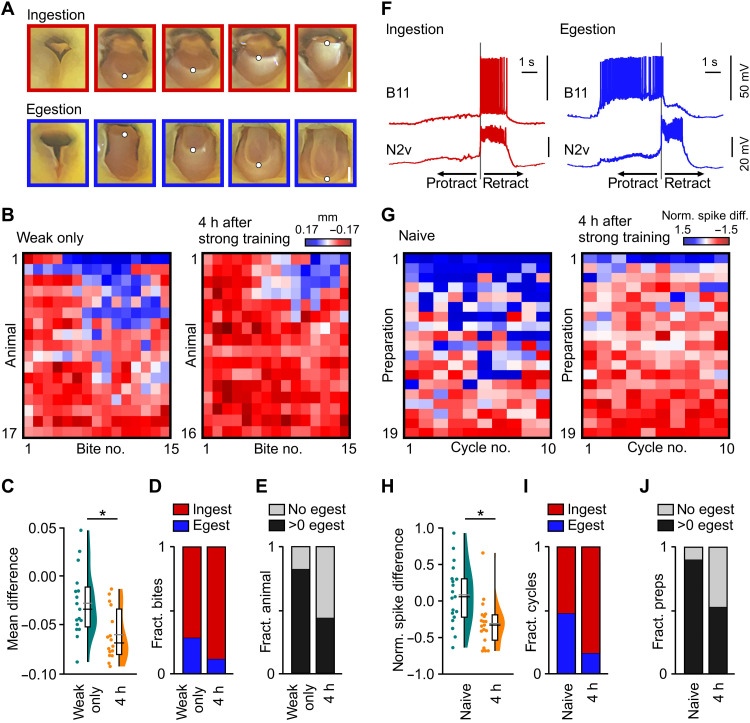
Previous learning alters the perception of future training by shifting the feeding network state. (**A**) Example frames showing mouth movements during ingestion or egestion. Frames are color-matched to (B) (red: ingestion, blue: egestion). White dots indicate distal tip of the radula tracked during bite classification. Scale bar, 0.5 mm. (**B**) Heat plots of radula movements during the first 15 bites in response to the CS + US during weak training in animals receiving weak training only versus animals receiving strong training 4 hours earlier. Red-white-blue lookup table represents radula movements. Positive (blue) is egestion; negative (red) is ingestion. (**C**) Statistical summary of (B) shows a significant change in the mean difference in radula movements between conditions (two-tailed *t* test, *P* < 0.01, *t* = 3.1). (**D**) Plot of fraction of ingestion/egestion bites produced during weak training showing a significant difference between conditions (Fisher’s exact test, *P* < 0.01). (**E**) Plot of fraction of animals performing no egestion bites versus >0 egestion bites showing a significant difference between conditions (Fisher’s exact test, *P* < 0.001). (**F**) B11 and N2v activity in an in vitro preparation during ingestion and egestion cycles. B11 is predominantly active in the retraction phase during ingestion and the protraction phase during egestion. N2v activity does not change during ingestion and egestion cycles. B11 is therefore a readout of ingestion versus egestion. Gray lines represent retraction phase onset. (**G**) Heat plots of B11 activity during fictive feeding cycles. Lookup table colors are normalized B11 spike differences. Positive (blue) is egestion; negative (red) is ingestion. (**H**) Statistical summary of (G) shows a significant change in B11 spike difference between conditions (Mann-Whitney test, *P* < 0.01, *U* = 70). (**I**) Plot of fraction of ingestion/egestion cycles showing a significant difference between conditions (Fisher’s exact test, *P* < 0.001). (**J**) Plot of fraction of preparations producing no egestion cycles versus >0 egestion cycles showing a significant difference between conditions (Fisher’s exact test, *P* < 0.001). h, hours.

How does prior strong learning enhance new memory formation? Given the strict order of training needed (strong followed by weak), we hypothesized that the strong memory might be changing the animal’s perception of the combination of the CS + US during subsequent weak training, increasing the positive value of the weak training such that the association becomes enhanced. To test this idea, we took advantage of the fact that the perception of a presented stimulus, from positive to negative, can be read out directly in *Lymnaea* by quantifying the number of ingestion and egestion events that occur (Fig. 2A) (*16*). During the pairing of the CS + US in the weak training protocol, animals performed a mixture of ingestion and egestion bites, often flip-flopping between bouts of the two behaviors (Fig. 2B). By contrast, when this was preceded by strong training, the ratio of ingestion to egestion was significantly higher (Fig. 2, C and D) and more animals performed no egestion bites at all in response to the weak training (Fig. 2E). Furthermore, to examine perceptual stability during weak training, we compared pairs of bites elicited during the CS + US presentation to determine whether they switched between states (ingest/egest and egest/ingest) or remained stable (ingest/ingest and egest/egest). This revealed a significant reduction in the transition probability between states, suggesting that prior strong training shifts, and stabilizes, the perception of the training (fig. S4, A to E). Presentation of the strong training US alone, 4 hours before weak training, did not cause any change in the ingestion/egestion behaviors compared to animals that did not receive the strong training US, demonstrating that the energetic value of high sucrose alone during strong training was not the source of the bias toward ingestion behavior (fig. S5, A to D). Furthermore, prior weak training (AA + s) 4 hours earlier also did not change the perception of subsequent weak training, confirming that the acquisition of a past strong memory is necessary to alter the perception of the weak training (fig. S5, A to D). We also ruled out the possibility that previous strong or weak training was simply altering the responsiveness to the CS or US used in the weak training (fig. S6, A to D). Together, our findings demonstrate that naïve animals perceive the CS + US used during weak training as an ambiguous/bistable stimulus and that previous strong training shifts the animal’s perception of the weak training to a stable state, biasing behavioral expression toward positive (ingestion) versus negative (egestion) behavior.

### Shifts in the perception of weak training are driven by changes in network state

We next set out to identify the neural changes that underlie the shift in perception of the CS + US used during weak training that is correlated with new memory acquisition. This is readily achievable in *Lymnaea* thanks to the highly accessible and well-defined neural circuits that control its feeding behavior. Specifically, we made intracellular recordings from the phase-switching feeding motoneuron, B11, which provides a readout of the relative activity in both the ingestion and egestion circuits and thus serves as an in vitro measure of the internal network state (Fig. 2F). In isolated brains, where sensory pathways were absent, we compared this readout in both naïve animals versus those that had previously received strong training. Matching our findings in vivo, preparations from naïve animals showed many rhythmic activity patterns known to underlie egestion cycles (fictive egestion), while those that had experienced strong training were almost exclusively biased toward the electrophysiological expression of ingestion cycles (fictive ingestion; Fig. 2, G to J, and fig. S7A). Furthermore, by examining pairs of cycles, we found that naïve preparations transition between ingestion and egestion cycles at a higher rate than preparations that have received the strong training (fig. S7, B to E). Preparations that received prior weak training showed no significant difference in their fictive motor programs compared to naïve preparations (fig. S7, F to K). This provides compelling evidence to suggest that strong training elicits a learning-induced shift and stabilization in the network state.

Next, we tested whether strong training altered the “responsiveness” of the feeding circuitry. To do this, we stimulated the medial lip nerve (MLN), a projection pathway carrying chemosensory input from the lip structures to the central nervous system (CNS) (*28*), which is known to drive strong fictive feeding (*29*). This stimulation does not encode specific information about the nature of the stimulus but activates all chemosensory projections, allowing us to test whether strong training induced a generalized sensitization effect regardless of the appetitive cue. We monitored this on B9, a motoneuron that provides a readout of feeding cycles in the circuit, and the cerebral giant cell (CGC), a key identified modulatory neuron that receives excitation from appetitive cues (*30*). We found that neither the number of feeding cycles nor the CGC spike frequency were significantly different between naïve and trained preparations (fig. S8, A to D) following lip nerve stimulation.

Together, these results demonstrate that strong training is not eliciting a generalized sensitization of the animal that enhances appetitive cue responses. Rather, there is a learning-induced shift in the network state after strong training that biases the network toward the generation of ingestion cycles. We propose that this underlies the altered perception that is observed in these animals, in turn enhancing their capability to acquire new memories.

### Elucidating the control circuit which drives the shift in perception

**Fig. 3.**
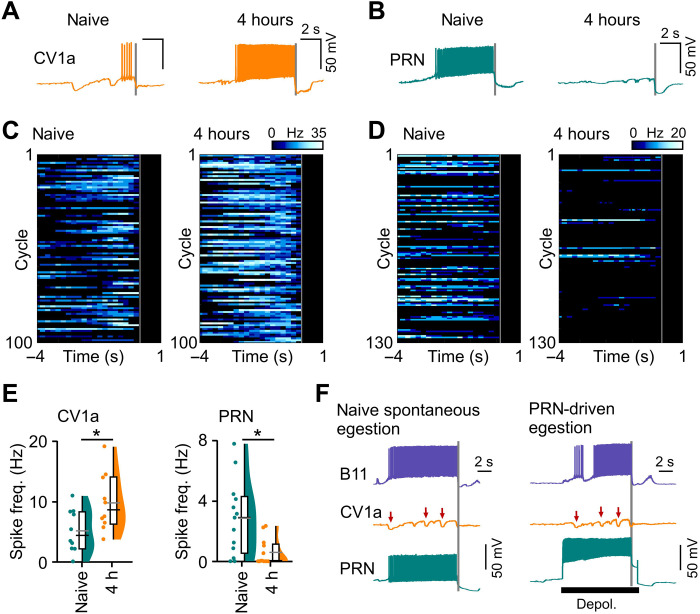
Learning-induced shift recorded in command-like interneurons controlling antagonistic behaviors. (**A** and **B**) Example traces showing activity of the ingestion command-like interneuron CV1a (A) and the egestion command-like interneuron PRN (B) during spontaneous cycles from naïve preparations and preparations from animals that received strong training 4 hours earlier. Gray lines represent retraction phase onset. (**C** and **D**) Heat plots of CV1a (C) and PRN (D) spike activity during multiple trials during in vitro cycles from naïve and trained preparations. Color code shows spike frequency. (**E**) Statistical analysis of (C) and (D). Four-hour trained preparations had more CV1a activity per cycle versus naïve (two-tailed *t* test, *P* < 0.05, *t* = 2.5), whereas PRN activity was significantly higher in naïve versus trained preparations (Mann-Whitney test, *P* < 0.01, *U* = 33.5). (**F**) Example traces of CV1a, PRN, and B11 activity during a spontaneous (left) and PRN-driven (right) egestion cycle. Red arrows indicate periods of inhibition on CV1a. h, hours.

Next, to characterize the circuit mechanisms that mediate this learning-induced shift in perception, we measured activity in two higher-order interneurons, cerebral ventral 1a (CV1a) and pattern reversing neuron (PRN). These cells are command-like neurons in the ingestion and egestion circuits, respectively, and, when active, are sufficient to drive their respective motor programs (*16*, *31*). Intracellular recordings showed that CV1a activity was significantly up-regulated at 4 hours after strong training versus naïve animals, while PRN activity was significantly down-regulated (Fig. 3, A to E). We reasoned that this learning-driven antagonistic activity relationship could be explained by a common circuit motif, namely, an inhibition between competing circuits (*32*). In this scenario, an up-regulation of activity in one network would serve to drive a suppression in the antagonistic one. In support of this, CV1a receives large inhibitory inputs throughout the protraction phase during both spontaneous and PRN-driven egestion cycles (Fig. 3F). These inhibitory inputs did not arise directly from PRN, suggesting that downstream neurons were recruited.

**Fig. 4.**
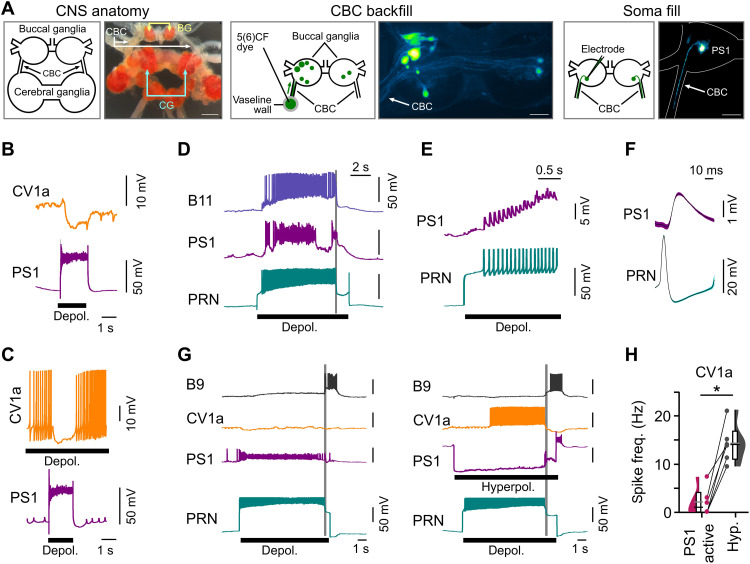
Characterization of perceptual control circuit that mediates competitive interactions between ingestion and egestion. (**A**) Identification of candidate egestion interneurons that inhibit ingestion. Left: Schematic and image of the *Lymnaea* central nervous system showing the main sites of the feeding circuitry. Middle: Strategy to reveal candidate buccal neurons projecting to cerebral ganglia (where CV1a is located) relies on back-filling the cerebrobuccal connective (CBC) with the fluorescent dye 5(6)-carboxyfluorescein. Right: Morphology and schematic of PS1 interneuron in the buccal ganglia after iontophoretic dye filling confirms single projection leaving ganglion via the ipsilateral CBC. Morphology was confirmed in *n* = 3 cells. Scale bars, 100 μm. (**B** and **C**) Artificial activation of PS1 hyperpolarizes membrane potential of CV1a (B) and can prevent spiking activity when CV1a is artificially depolarized to tonically spike (C). (**D**) PS1 is strongly activated during a PRN-driven egestion cycle, corecorded with B11. Gray lines represent retraction phase onset. (**E** and **F**) Monosynaptic excitatory connection between PRN and PS1; evoked spikes in PRN excite PS1 and generate 1:1 EPSPs (F). (**G**) Representative traces of a PRN-driven egestion cycle. PS1 is strongly recruited, and CV1a shows no spiking activity (left). Hyperpolarizing PS1 throughout the PRN-driven cycle causes CV1a to be strongly recruited during the protraction phase (right). (**H**) Summary plot. CV1a spike frequency was significantly greater during PRN-driven cycles where PS1 was artificially hyperpolarized (*n* = 6, two-tailed paired *t* test, *P* < 0.001, *t* = −7.2).

Next, to identify the source of these inputs, we carried out an extensive search for a neuron type that would fulfil the following criteria: (i) It should inhibit CV1a when active, (ii) it should be active during the protraction phase of egestion cycles, and (iii) it should be excited by PRN activity. Using a fluorescence labeling approach to reveal neurons projecting from the buccal ganglia where most of the feeding circuitry is housed (*33*), we identified a single candidate neuron type, pattern switch 1 (PS1) (Fig. 4A), that satisfied all three criteria. First, artificial stimulation of PS1 monosynaptically inhibited the ipsilateral CV1a (Fig. 4, B and C). Second, this neuron was strongly active during the protraction phase of both PRN-driven and stimulus-driven egestion cycles (Fig. 4D and fig. S9A) when CV1a is inhibited. Third, PRN activity excited PS1 monosynaptically, observed as 1:1 excitatory postsynaptic potentials (EPSPs) (Fig. 4, E and F). To ascertain whether PS1 was the sole source of inhibition to CV1a during egestion feeding behavior, we artificially manipulated its activity during PRN-driven cycles. When hyperpolarized, there was now a significant increase in CV1a activity (Fig. 4, G and H) with no evidence for the phasic inhibitory synaptic inputs it normally receives. Moreover, PS1 hyperpolarization was also sufficient to increase CV1a activity during sensory-driven egestion cycles (fig. S9B). Thus, this pivotal inhibitory neuron type acts as a switch during action selection, preventing disruptive activation of the ingestion command centers during egestion.

Since CV1a does not have a monosynaptic connection with PRN, how does it ensure the suppression of egestion when it is active? We were able to answer this question by identifying the second component of the control circuit: a buccal interneuron type, PS2, which was strongly electrically coupled to PRN and sufficient to drive robust egestion cycles (fig. S9, C and D). It receives strong facilitating inhibition from CV1a and thus leads to the suppression of egestion when ingestion cycles are generated (fig. S9, E and F). Moreover, artificial activation of PS2 caused polysynaptic inhibitory inputs on CV1a, arising through its monosynaptic excitatory connection onto PS1 (fig. S9, G and H). Together, these results demonstrate that mutual inhibition is used to prevent coactivation of competitive circuits and that this circuit motif provides a control point on which plasticity could act (fig. S9I). Next, we explored the possible role for suppression of the egestion circuit in biasing the perception of learning events.

### Manipulation of perceptual control circuit enables new learning in vivo

**Fig. 5.**
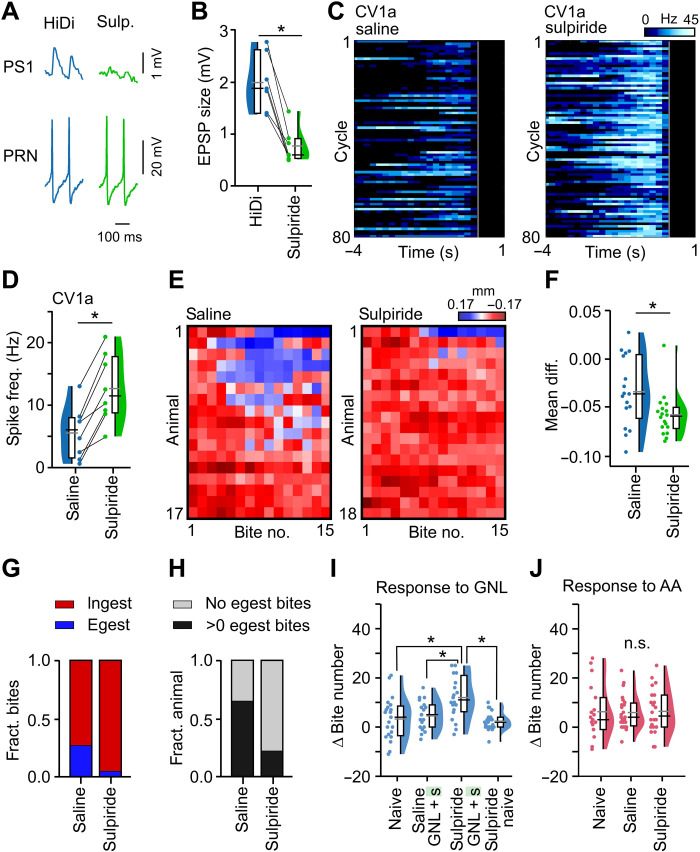
Pharmacological manipulation of perceptual control circuit substitutes for strong training and facilitates new memory acquisition in vivo. (**A**) Sulpiride (10^−4^ M) reduces EPSP amplitude in PS1 arising from PRN spikes. High divalent (HiDi) saline was used to reveal monosynaptic connections. (**B**) EPSP size was significantly reduced by sulpiride (*n* = 7, Wilcoxon test, *P* < 0.05, *W* = 28). (**C**) Heat plots of CV1a activity during in vitro cycles from naïve preparations in saline and sulpiride. Gray lines represent retraction phase onset. (**D**) CV1a activity was significantly higher in sulpiride versus saline (*n* = 8, two-tailed paired *t* test, *P* < 0.001, *t* = −7.2). (**E**) Sulpiride shifts perception of weak training. Heat plots of radula movements during the first 15 bites in response to the CS + US during weak training in saline or sulpiride-injected animals. (**F**) Summary analysis of (E) showing a significant shift toward ingestion bites in sulpiride versus saline-injected animals (Mann-Whitney test, *P* < 0.05, *U* = 81). (**G**) Comparison of fraction of ingestion/egestion bites in response to the weak training showing a significant difference between conditions (Fisher’s exact test, *P* < 0.001). (**H**) Comparison of fraction of animals performing no egestion bites versus those performing >0 egestion bites in response to weak training shows a significant difference between conditions (Fisher’s exact test, *P* < 0.001). (**I**) Sulpiride injection 2 hours before weak training significantly increases the response to the CS (tested 1 day later) versus naïve animals, saline-injected weak trained animals, and sulpiride-injected naïve animals {naïve, *n* = 21; saline and weak trained, *n* = 19; sulpiride and weak trained, *n* = 20; sulpiride only, *n* = 22; one-way ANOVA, *P* < 0.001 [*F*(3,78) = 8.3]; Tukey’s test: sulpiride and weak trained versus naïve, *P* < 0.001, versus saline and weak trained, *P* < 0.01, versus sulpiride only, *P* < 0.001; all other conditions, *P* > 0.05}. (**J**) Sulpiride injected 2 hours earlier produced no significant increase in response to AA versus saline-injected and naïve animals {naïve; *n* = 19, saline-injected; *n* = 24, sulpiride-injected; *n* = 22, one-way ANOVA, *P* > 0.05 [*F*(2,62) = 0.015]}.

Given that it favors the expression of egestion behavior, we reasoned that a reduction in PRN → PS1 activity might be sufficient to alter an animal’s perception of weak training and thus enhance memory acquisition. To investigate this, we developed a pharmacological strategy that allowed us to manipulate the PRN output pathway. We have previously shown that this neuron is dopaminergic and that the D2 receptor blocker, sulpiride, is highly effective in inhibiting its action on follower motoneurons (*16*). Here, we confirmed that the PRN → PS1 connection is also sulpiride sensitive (Fig. 5, A and B), causing a significant reduction in the amplitude of the PRN → PS1 EPSP. Next, we tested whether blocking this connection could mimic the increase in CV1a activity observed 4 hours after strong training. We found that sulpiride caused a robust elevation in CV1a cycle activity versus pretreatment (Fig. 5, C and D), consistent with our previous work showing that sulpiride application biases activity toward ingestion events (*16*). Thus, sulpiride can robustly alter the network state, substituting for the effects of strong training. As such, this agent provides the opportunity to test in vivo whether this pathway underlies the animal’s altered perception of the weak training. Animals were injected with either sulpiride or normal saline and underwent the weak training protocol with feeding responses measured as in Fig. 2 (A and B). We found that sulpiride-injected animals performed significantly more ingestion events in response to the weak training than saline-injected animals (Fig. 5, E to G), and significantly more of the sulpiride-injected animals performed no egestion responses at all (Fig. 5H). Moreover, there was a significant decrease in the transition probability between states after sulpiride injection (fig. S10, A to D). Therefore, both strong training and sulpiride injection shift the network state in vitro and stabilize the perception of weak training in vivo.

We next tested whether the shift in perception induced by sulpiride was sufficient for acquisition of LTM after weak training, as we have shown in the case of strong training. Animals were injected with sulpiride or saline and then underwent weak training with LTM tested 1 day later. Consistent with the effects of strong training, we found that animals injected with sulpiride before weak training had a significantly greater response to GNL compared to naïve or saline-injected trained animals (Fig. 5I). Furthermore, sulpiride injection in the absence of weak training did not increase the feeding response to GNL when tested 1 day later (Fig. 5I). Therefore, pharmacologically manipulating the network state causes a change in the perception of the weak training that is sufficient for the animal to acquire and fully consolidate a memory.

Next, we examined whether the identified learning-induced change in network state after strong training was involved in the expression of the original strong memory or alternatively whether this was a parallel process that served the purpose of enhancing future learning events. To test this, we injected animals with sulpiride or saline and recorded their response to the strong training CS (AA) but in the absence of prior strong training. We hypothesized that if the learning-induced change in network state was involved in the expression of the original memory, then feeding behavior in response to the CS (AA) used for strong training would be increased by artificially inducing the same change in network state with sulpiride, but in the absence of prior strong training. However, we found that sulpiride injection did not cause an increase in the response to AA compared to naïve or saline-injected animals (Fig. 5J). Therefore, although the strong training causes a shift in the network state, this learning-induced change does not actively participate in the expression of the strong memory itself, suggesting that distinct mechanisms are involved. Together, these results demonstrate that strong learning causes parallel changes in neural activity: one for the expression of the memory itself and one for altering the perception of future appetitive learning.

### Memory-linked shifts in perception generalize to an alternative training paradigm

**Fig. 6.**
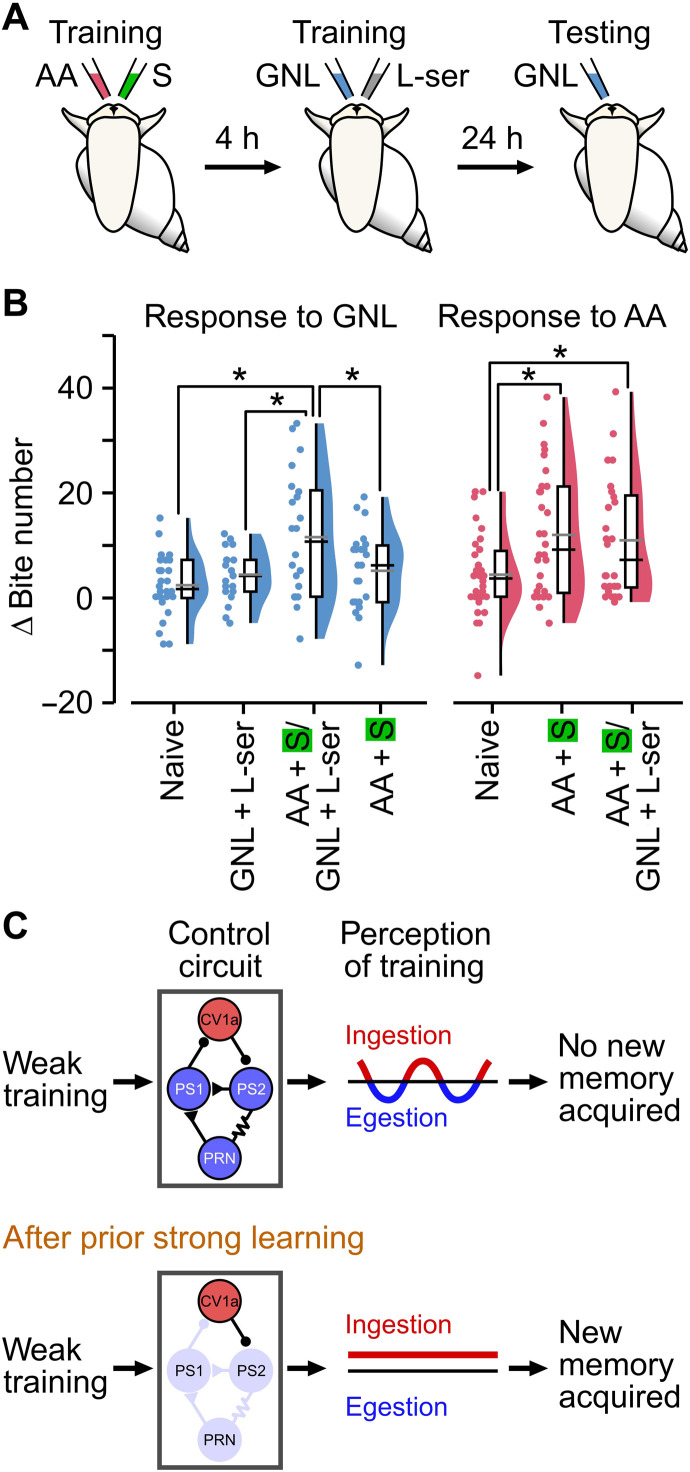
Memory-linked shifts in perception generalize to an alternative training paradigm. (**A**) Cartoon of double training paradigm using a distinct CS + US for each training. Strong training consisted of pairing AA as the CS with sucrose (0.33%) as the US. Four hours later, animals received weak training of GNL as the CS paired with l-serine (0.11%) as the US. One day later, animals were tested for their CS response. (**B**) Strong training 4 hours before weak training with l-serine as the US led to a significant increase in the response to GNL versus naïve, weak only, or strong only {naïve, *n* = 26; weak only, *n* = 19; strong 4 hours before weak, *n* = 22; strong only, *n* = 24; one-way ANOVA, *P* < 0.05 [*F*(3,87) = 5.8]; Tukey’s test: strong 4 hours before weak versus naïve, *P* < 0.001, versus weak only, *P* < 0.05, versus strong only, *P* < 0.05; all other conditions, *P* > 0.05}. Strong training followed by weak training also resulted in a significantly increased response to AA versus naïve, but not strong only {naïve, *n* = 32; strong only, *n* = 30; strong 4 hours before weak, *n* = 26; one-way ANOVA, *P* < 0.01 [*F*(2,85) = 5.02]; Tukey’s test: naïve versus strong only, *P* < 0.05; naïve versus strong 4 hours before weak, *P* < 0.05; strong only versus strong 4 hours before weak, *P* > 0.05}. (**C**) Schematic summarizing the proposed mechanism for how a learning-induced shift in network state could alter the perception of subsequent weak training and enhance new memory acquisition. Weak training alone is perceived as ambiguous/bistable due to the competitive interactions between the ingestion-driving and egestion-driving interneurons, resulting in the animal transitioning between the two behaviors with no resulting memory acquisition. Prior strong training, however, stabilizes the network state and biases activity toward the ingestion circuit, shifting the perception of subsequent weak training and enhancing new memory acquisition. h, hours.

What role does the mechanism identified here serve? The ability to link strong and weak learning events that are closely temporally coupled suggests that *Lymnaea* could use this capability to identify “learning-rich” time periods, for example, compatible with the arrival of the animal in a bountiful environment. If so, we would expect that learning should generalize rather than depend on the same US for both the strong and weak training. To test this important idea, we carried out experiments in which we substituted the sucrose US used in the weak training protocol with l-serine (Fig. 6A), a known alternative appetitive stimulus in *Lymnaea* (*29*). Thus, both the CS and US are distinct across the two different training paradigms. We found that while the GNL + l-serine pairing alone did not yield a conditioned response, a robust 1-day memory expression to GNL was seen if the strong training preceded it (Fig. 6B). Thus, memory expression is not confined to one US but can instead enable the formation of different associations, suggesting it would be highly appropriate for enabling a generalized lowering of the threshold for forming new memories.

## DISCUSSION

The ability to learn new associations is critical for survival in an unpredictable environment. Given that memory acquisition and consolidation are energetically costly processes (*1*, *2*, *34*), there are key potential benefits in using strategies that help guide decisions about what and when to learn. Here, we identify a simple mechanism in *Lymnaea* by which past events are used to bias perception. Notably, we also demonstrate that this mechanism can guide future learning—facilitating memory acquisition for associations that would previously have been ignored—and we elucidate the neural circuits responsible. We propose that this serves to alert an animal to efficiently direct resources to new learning where recent experience suggests that there may be a particular advantage in forming positive associations.

A key feature of the mechanism we elucidate is that strong learning initiates parallel processes in the brain: one for the expression of the original memory and one to alter the perception of future learning events and facilitate new memory acquisition. These appear to be independent since the shift in neural network state measured after strong training is not sufficient for the expression of the original memory, as evidenced by the absence of a false memory when the network state is changed pharmacologically. Similar parallel pathways have been identified in conditioning experiments in mice. For example, in an olfactory-discrimination task, extensive training changed the excitability of pyramidal neurons in the piriform cortex coinciding with the enhanced capability to learn new tasks (rule learning), but this did not correlate with the expression of the original memory (*35*, *36*). As such, while these learning-induced changes are not part of the memory-expressing “engram,” they nonetheless serve critical adaptive functions in animals, allowing them to use their past experiences to guide their future behavior—a process arguably as important as recall of the memory itself.

The mechanism that we have identified exhibits key timing characteristics. It permits enhanced learning capabilities from 30 min up to 4 hours after strong training, suggesting that new memory acquisition is facilitated in a critical time window. Informed by our previous work, this time frame aligns with the emergence of intermediate-term memory for the strong training and lasts until the emergence of LTM (*17*). The expression of this memory over this time frame is protein synthesis dependent, while the memory trace recorded at a shorter, 10-min time point is not (*17*). This strongly suggests that the shift in network state identified in our present study is also protein synthesis dependent, explaining the absence of an effect at 10 min after strong training. Furthermore, the enhanced learning capability is not maintained at longer time points: Weak learning memory formation is absent from ~6 hours onward, although the original strong memory is still present and expressible. Therefore, the learning-induced shift in network state is transiently induced and does not outlast the molecular mechanisms known to be necessary for consolidation of the original memory during the first 6 hours after strong training (*37*). We hypothesize that a permanent shift in perception due to past learning could be detrimental to the animal, leading to energetically costly and potentially maladaptive memories. There is also a requirement for a strict temporal sequence of strong followed by weak training. This differs from a previously identified process, behavioral tagging (*38*, *39*), which facilitates the interaction and enhancement of memories based on synaptic tag and capture (*40*, *41*). During behavioral tagging, a tag set in motion by a weak learning event is targeted and enhanced by a strong learning event regardless of the temporal sequence of the two learning events (*42*, *43*). This key feature of behavioral tagging leads to an alteration of the memory of the event, not the learning event itself. By contrast, the learning-induced shift in perception we identify here guides the animal to decide which future events to learn about, rather than which recently acquired memories should be further consolidated. As such, while the two mechanisms have some parallels and both serve to increase the number of consolidated long-term memories, they function under different circumstances and use distinct circuit mechanisms. We also demonstrate that the learning-induced shift in perception generalizes to other forms of appetitive learning, since a second type of weak appetitive training could also be enhanced by past strong learning. A similar generalized enhancement of learning capabilities has been found in mice, where hippocampus-dependent olfactory-discrimination learning is thought to switch the hippocampal network into a “learning mode,” enhancing other types of hippocampus-dependent learning, such as spatial learning (*44*). The time course and nonspecific nature of the mechanism identified in *Lymnaea* could serve to alert the animal to a “learning-rich” time period, permitting positive associations to be made to cues which, if encountered in isolation, would likely be disregarded.

What mechanism facilitates weak training after strong training? We note that naïve animals exposed to the weak training “flip-flop,” or transition, between ingestion and egestion behaviors, suggesting that the animal has a bistable perception of the CS + US stimuli during the training, which represents a period of ambiguity (*45*). Notably, we also find that the strong training biases and stabilizes the animal’s perception of the weak training to favor ingestion behavior. We suggest that bistable perception during weak training is an emergent property of the relative activity of the ingestion and egestion circuits since the same switching persists in vitro in the absence of any external stimulation. By characterizing the connectivity between the two circuits, we show that competitive interactions are due to a reciprocal inhibition circuit motif (*32*, *46*), suggesting that action selection is likely generated by a winner-takes-all model. Feedforward inhibition from the egestion circuit prevents coactivation of the ingestion circuit (PRN + PS2 → PS1 → CV1a), whereas the ingestion circuit directly inhibits part of the electrically coupled egestion circuit (CV1a → PS2) (Fig. 6C and fig. S9I). Since both circuits are innervated by the same core feeding central pattern generator (*16*, *31*), we propose that cycle expression is dictated by the first circuit to reach a sufficient spike-rate threshold to inhibit the other. After strong training, there is a shift in the relative activity of the two circuits and, as such, expression of ingestion cycles is dominant (Fig. 6C). A similar circuit motif is seen during fear conditioning in mice, where one of two incompatible behaviors can be generated: escape or freezing. Learning shifts the balance of two reciprocally inhibitory classes of neurons in the central amygdala, which biases expression toward one or the other behavior (*47*). In *Lymnaea*, however, the learning-induced shift in activity is not used for expression of the memory but rather for the facilitation of new learning (Fig. 6C). We show that pharmacologically stabilizing the ingestion circuit by blocking the feedforward inhibition from the egestion circuit is sufficient to alter the animal’s perception of the CS + US during weak training in vivo. Furthermore, we show that this can substitute for the acquisition of a strong memory, shifting the network state to enable new positive associations, suggesting that memory formation can be facilitated by reducing ambiguity during a learning event. Previous studies in humans have demonstrated that prior learning can alter attentional control, which tunes perceptual sensitivity (*3*–*5*), and attention has also been shown to have a role in perceptual multistability (*45*, *48*). Although only the animal’s perceptual readout was measured in this study, it is possible that the mechanism identified here also includes an attentional shift that could modulate the perception of the weak training. The fact that the shift in network state is present before exposure to the weak training could suggest that there is an anticipatory attention mechanism that, in turn, tunes the perception of future learning events. However, if such an attentional shift is involved, it is apparently only able to modulate perception and, thus, enhance new memory formation from ~30 min after the strong training.

Why does the stabilization of the network toward ingestion events facilitate weak training? One simple explanation is that, during ingestion, the animal actively draws the CS and US into the buccal cavity and then the esophagus, allowing each to be swallowed. This contrasts with egestion where contents are efficiently expelled from the buccal cavity. Earlier work has demonstrated that successful in vitro conditioning depends on the US reaching the esophagus (*17*) and activating esophageal neurons (*49*), which reinforce the CS via D1 receptor activation in follower neurons (*50*–*52*). Therefore, favoring ingestion cycles will serve to increase the US reaching and activating these neurons and thus reinforcing the CS + US.

Here, we have uncovered a so far unreported mechanism by which changes in perception can couple prior and new learning under specific conditions and time frames. We propose that this can serve as a general state setting mechanism, allowing an animal to form associations between new combinations of stimuli, which, in isolation, would be insufficient to induce memory. In view of the fact that associations between learning and perceptual changes are well established in higher animals, including humans, we suggest that a learning → perception → learning pathway may be a broadly conserved feature that deserves further attention in learning studies. Given that the formation of LTM is associated with increased energetic costs—particularly the engagement and recruitment of molecular machinery for memory consolidation—a mechanism that directs learning has potentially important value for survivability. In the case of a foraging animal such as *Lymnaea*, operating on a tight energy budget (*53*), learning different associations is clearly highly beneficial, alerting them to potential food sources or possible dangers in their environment, but this needs to be balanced against the energetic costs of consolidating those memories. This mechanism thus allows them to tune their future learning to past learning success, by effectively lowering the threshold needed to learn new associations.

## MATERIALS AND METHODS

### Animal maintenance

Animals were kept in groups in large holding tanks containing Cu^2+^-free water at 20°C on a 12-hour light/12-hour dark regime. The animals were fed lettuce three times a week and a vegetable-based fish food (Tetra-Phyll; TETRA Werke, Melle, Germany) twice a week. Animals were transferred to smaller holding tanks and food-deprived for 2 days before experiments. For all the experiments, adult (3 to 4 months old) snails (*Lymnaea stagnalis*) were used. *Lymnaea* is a lower invertebrate (molluscan) organism that does not fall under The Animals (Scientific Procedures) Act 1986 (UK). Therefore, no ethical approval or guidance was required for these experiments.

### Single-trial appetitive training and testing procedures

Strong single-trial appetitive conditioning was performed by pairing AA (0.004%) as the CS with sucrose (0.33%) as the US using a previously well-described method (*23*, *24*). Weak single-trial appetitive conditioning was performed by pairing GNL (0.004%) as the CS with sucrose (0.11%) or l-serine (0.11%) as the US. In a counterbalance control experiment, strong conditioning was performed by pairing GNL as the CS with sucrose (0.33%) as the US, and weak conditioning was performed by pairing AA as the CS with sucrose (0.11%) as the US. Briefly, animals were placed individually in petri dishes containing 90 ml of Cu^2+^-free water for 10 min to acclimatize to the new environment before the training procedure began. Five milliliters of the CS was added to the water, and 30 s later, 5 ml of the US was applied. Animals were left in the solution containing the CS and US for 2 min, then rinsed in Cu^2+^-free water, and returned to their home tanks. To test for CS responses 1 day after conditioning, animals from trained and naïve groups were transferred from their home tanks to a petri dish filled with 90 ml of Cu^2+^-free water and allowed to acclimatize for 10 min. Five milliliters of water was then added to the dish and the number of feeding responses (bites) in the following 2 min was counted. Next, 5 ml of the CS was added to the dish, and the number of feeding responses was counted in the subsequent 2 min. CS responses were then assessed using a “difference score” (∆ bite number). This was obtained by subtracting the number of feeding cycles observed during 2 min after water application from the number of feeding cycles in 2 min after CS application. During dual conditioning experiments, animals received both the strong and weak appetitive conditioning separated by time intervals as described in the results. To test whether preexposure to the US used during strong training (0.33% sucrose) enhanced weak training learning, we performed the strong training as described above but in the absence of presentation of the CS (AA). Animals then received the weak training of GNL and sucrose (0.11%) 4 hours later and were tested for their response to GNL 1 day later.

### Measurement of perception during weak training

The effect of past learning on the animal’s perception of weak training was tested by performing strong training followed by weak training 4 hours later as above. During the weak CS + US presentation, animals’ feeding responses were videoed (33 frames/s) from below. The direction of movement of the radula and underlying odontophore structure was measured during the first 15 feeding responses as in (*16*). Briefly, the position of the dorsal mandible was first marked in the frame preceding the first frame in which the radula was visible during each bite using ImageJ software. The radula was then tracked for the entire bite, and the distance from the initial position of the dorsal mandible was calculated. The average difference moved between frames was then measured. A negative score therefore represented the radula and dorsal mandible being apart at the start of the bite and the radula moving toward the dorsal mandible during the bite, whereas a positive score represented the mandible and radula being close together at the start of the bite and the radula moving away as the bite progressed. The criteria to define whether a response was ingestion or egestion were based on whether the difference in movement was negative (ingestion) or positive (egestion). To measure how prior strong training alters the stability of the perception of the CS + US used in weak training, pairs of consecutive bites were analyzed. A stable bite pair was classified as two of the same consecutive bites (ingest-ingest or egest-egest). A switching bite pair was classified as two different consecutive bites (ingest-egest or egest-ingest). Transition probabilities were then calculated by counting the number of switching bites expressed as a fraction of the total number of bite pairs. To test whether preexposure to the US used during strong training (0.33% sucrose) altered the perception of weak training, we performed the strong training as described above but in the absence of presentation of the CS (AA) and measured ingestion/egestion behaviors as above. To test whether prior weak training altered the perception of later weak training, animals first received AA paired with 0.11% sucrose and then their ingestion/egestion responses to GNL and 0.11% sucrose were measured 4 hours later. To test for the effects of strong training on the responsiveness of the animal to either the CS or US used during weak training, animals received the strong training as above. Four hours later, animals were placed in a petri dish of 90 ml of Cu^2+^-free water and allowed to acclimatize for 10 min. They then received 5 ml of water, and their feeding responses were counted. They subsequently received 5 ml of either GNL or 0.11% sucrose, and feeding responses were counted so that the ∆ bite number could be calculated as above.

### Preparations and electrophysiological methods

Following procedures previously described in (*16*), we carried out in vitro experiments using an isolated CNS preparation. A small region of the anterior esophagus was kept attached to the CNS by the dorsal buccal nerves. Preparations were perfused with normal saline containing 50 mM NaCl, 1.6 mM KCl, 2 mM MgCl_2_, 3.5 mM CaCl_2_, and 10 mM Hepes buffer in water. Monosynaptic connections were tested for by bathing the preparation in a high divalent (HiDi) saline, which increases action potential threshold, reducing polysynaptic connections. HiDi saline was composed of 35.0 mM NaCl, 2 mM KCl, 8.0 mM MgCl_2_, 14.0 mM CaCl_2_, and 10 mM Hepes buffer in water. Intracellular recordings were made using sharp electrodes (10 to 40 megohms) filled with 3 M KAc and 0.5 mM KCl. Signals were collected using NL 102 (Digitimer Ltd.) and Axoclamp 2B (Axon Instrument, Molecular Device) amplifiers, and data were acquired using a micro 1401 Mk II interface and analyzed using Spike2 software (Cambridge Electronic Design, Cambridge, UK).

### Neuron identification

The phase switching motoneuron B11 is located in the buccal ganglia and was identified on the basis of its location, spike shape, synaptic inputs from PRN, and ability to switch its activity pattern during ingestion and egestion cycles (*16*). The ingestion command-like interneuron CV1a is located in the cerebral ganglia and was identified by its electrical properties, characteristic location, and its ability to drive fictive feeding cycles when artificially depolarized to fire spikes (*31*). The egestion command-like interneuron PRN is located in the buccal ganglia and was identified by its location and monosynaptic excitatory connection to B11. The N2v neuron is a central pattern generator interneuron located on the ventral surface of the buccal ganglia. It can be identified by its characteristic plateau during the retraction phase of a cycle, and artificial activation causes widespread retraction phase activity in many buccal neurons (*25*). B9 is a retraction phase motoneuron located in the buccal ganglia. The CGCs are large, serotonergic interneurons located in the cerebral ganglia, which can be identified by their size, location, and tonic spiking activity (*30*). To identify previously uncharacterized candidate members of the egestion network, we backfilled the cerebrobuccal connective (CBC) with the fluorescent dye, 5(6)-carboxyfluorescein (5-CF). Projection interneurons are known to be influential in driving patterned activity in *Lymnaea* (*30*, *31*), and egestion driving neurons have been identified in the buccal ganglia (*16*). Backfilling the CBC revealed a population of buccal projection interneurons that we could reidentify in other preparations and test electrophysiologically. Neurons of interest were impaled and corecorded with ingestion and egestion command-like neurons.

### Analysis and classification of in vitro cycles

Activity on B11 was measured with respect to the onset of the retraction phase, as determined by the N2v plateau or large excitation on retraction phase interneuron B9. To analyze the relative activity of B11 in a cycle, it was measured 4 s before and 4 s after retraction phase onset. The number of B11 spikes after retraction phase onset was subtracted from the number of B11 spikes before retraction phase onset and then divided by the total number of spikes in the 8-s period to gain a normalized difference score. Using this score, a positive value represents more activity occurring before the retraction phase onset and therefore is classified as an egestion cycle. A negative score represents more activity occurring after retraction phase onset and is therefore classified as an ingestion cycle. To compare the effects of strong training on fictive feeding cycles in vitro, the first 10 spontaneous cycles were analyzed from 19 naïve and 19 trained preparations. To measure how training alters the stability of cycle expression, pairs of consecutive cycles were analyzed. A stable cycle pair was classified as two of the same consecutive cycles (ingest-ingest or egest-egest). A switching cycle pair was classified as two different consecutive cycles (ingest-egest or egest-ingest). Transition probabilities were then calculated by counting the number of switching cycles expressed as a fraction of the total number of cycle pairs. To test whether strong training alters the responsiveness of the preparation to appetitive cues, we stimulated the main chemosensory pathway, the MLN (*28*), which can drive strong fictive feeding (*29*). The MLN was stimulated using a glass suction electrode with biphasic pulses of 4 V with 0.5-ms duration at 1 Hz for 120 s. The ∆ fictive feeding cycle number was calculated by recording activity in feeding motoneurons, such as B9, counting the number of cycles that occurred in the 120-s period preceding MLN stimulation, and subtracting this from the number of cycles in response to MLN stimulation. CGC activity was measured for the 120 s before and 120 s during the MLN stimulation. To elicit sensory-driven egestion in vitro, a 1-s tactile stimulus was applied to the esophagus, which activates mechanosensory neurons that signal aversive cues to the feeding network in response to overextension of the gut due to an inedible object lodged in the esophagus (*16*). The tactile stimulus was applied using a mechanical probe controlled by a transistor–transistor logic pulse from the micro 1401 Mk II (CED).

### Iontophoretic dye filling of neurons

Following procedures previously described in (*16*), we filled target neurons with a fluorescent dye (5-CF) using a microelectrode. This was achieved iontophoretically using a pulse generator to apply regular interval negative square current pulses into the neuron for >30 min. Preparations were then left overnight at 4°C. Images of the neurons were taken using a digital camera (Andor Ixon electron multiplying charge-coupled device) mounted on a Leica stereomicroscope.

### D2 receptor blocker application in vitro and in vivo

Sulpiride is an effective dopamine antagonist in *Lymnaea*, blocking the effects of dopaminergic interneurons on follower neurons as well as the focal application of dopamine (*16*, *54*). To test for the effect of sulpiride (±) (Sigma-Aldrich) on the PRN → PS1 connection, preparations were first bathed in HiDi saline (see above). Baseline EPSP amplitudes were recorded before 10^−4^ M sulpiride in HiDi saline was perfused into the bath for 10 min and then EPSP amplitudes were recorded again. To test the effects of sulpiride on in vitro cycle generation in naïve preparations, the first 10 spontaneous cycles generated were recorded and then 10^−4^ M sulpiride in normal saline was perfused onto the preparation. The first 10 spontaneous cycles generated after 10 min of perfusion were analyzed. To test the effects of sulpiride on the perception of weak training and memory acquisition/recall, animals were injected with 100 μl of 10^−3^ M sulpiride in normal saline. It has previously been shown that the injected concentration of the drug is diluted ~10-fold by the body fluids of the animal (*55*). Control animals were injected with 100 μl of normal saline alone. Animals were left for 2 hours before behavioral tests were carried out.

### Data analysis

Data were analyzed using paleontological statistics (PAST version 4.1) (*56*) and expressed as raincloud plots (*57*). In all cases, individual points are plotted as dots, and the shaded region (cloud) indicates the overall shape of the distribution extending from minimum to maximum values. Internal boxplots show median (black line) and interquartile range (first and third quartile) and mean (gray line). Each “*n*” represents an individual animal/preparation. Normality was tested using the Shapiro-Wilk test. Two-group statistical comparisons were performed using two-tailed *t* test statistics (either paired or unpaired as stated in the text) or a Mann-Whitney test or Wilcoxon signed-rank test for nonparametric data. Data with more than two groups were first analyzed using a one-way analysis of variance (ANOVA) or Kruskal-Wallis test. Subsequent comparisons were performed using Tukey’s or Dunn’s post hoc tests with Bonferroni sequential correction. The comparisons between the percentage of bites/cycles classified as ingestion or egestion and number of animals/preparations performing zero or more than zero egestion cycles were made using a Fisher’s exact test. The significance level was set at *P* < 0.05.

## Notes

View/request a protocol for this paper from *Bio-protocol*.
