# Supplementary material for: A circuit mechanism linking past and future learning through shifts in perception
Source: Sci Adv. 2023 Mar 24;9(12):eadd3403. doi: 10.1126/sciadv.add3403 (PMC10038338; doi:10.1126/sciadv.add3403)
Supplement: Supplementary file 1 — Figs. S1 to S10 Legend for movie S1 [file sciadv.add3403_sm.pdf]

Science Advances
AAAS

Supplementary Materials for

A circuit mechanism linking past and future learning through shifts in perception

Michael Crossley *et al.*

Corresponding author: Kevin Staras, k.staras@sussex.ac.uk; Michael Crossley, m.crossley@sussex.ac.uk

Sci. Adv. **9**, eadd3403 (2023)
DOI: 10.1126/sciadv.add3403

The PDF file includes:

Figs. S1 to S10
Legend for movie S1

Other Supplementary Material for this manuscript includes the following:

Movie S1

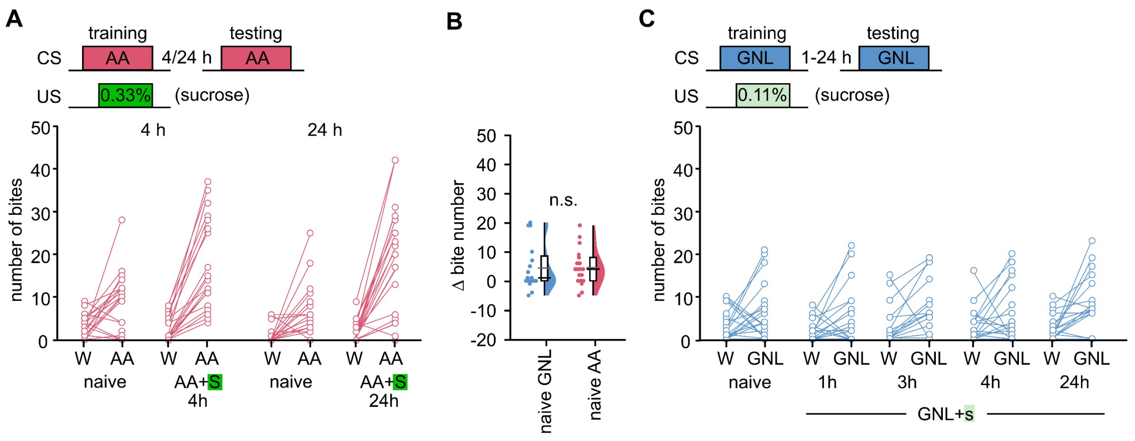

Fig. S1. Response to the two conditioned stimuli, AA and GNL, after single-trial appetitive conditioning.

(**A**) Presentation of data showing the bite numbers in response to water and AA used to calculate the Δ bite number in Figure 1B. Schematic of timeline of experiment (top).

(**B**) AA and GNL did not differ in their effect as a neutral stimulus in naïve animals. There was no significant difference in Δ bite number in naïve animals in response to GNL (n=17) or AA (n=19, Mann Whitney test, $p > 0.05$, U = 145).

(**C**) Presentation of data showing the bite numbers in response to water and GNL used to calculate the Δ bite number in Figure 1C. Schematic of timeline of experiment (top).

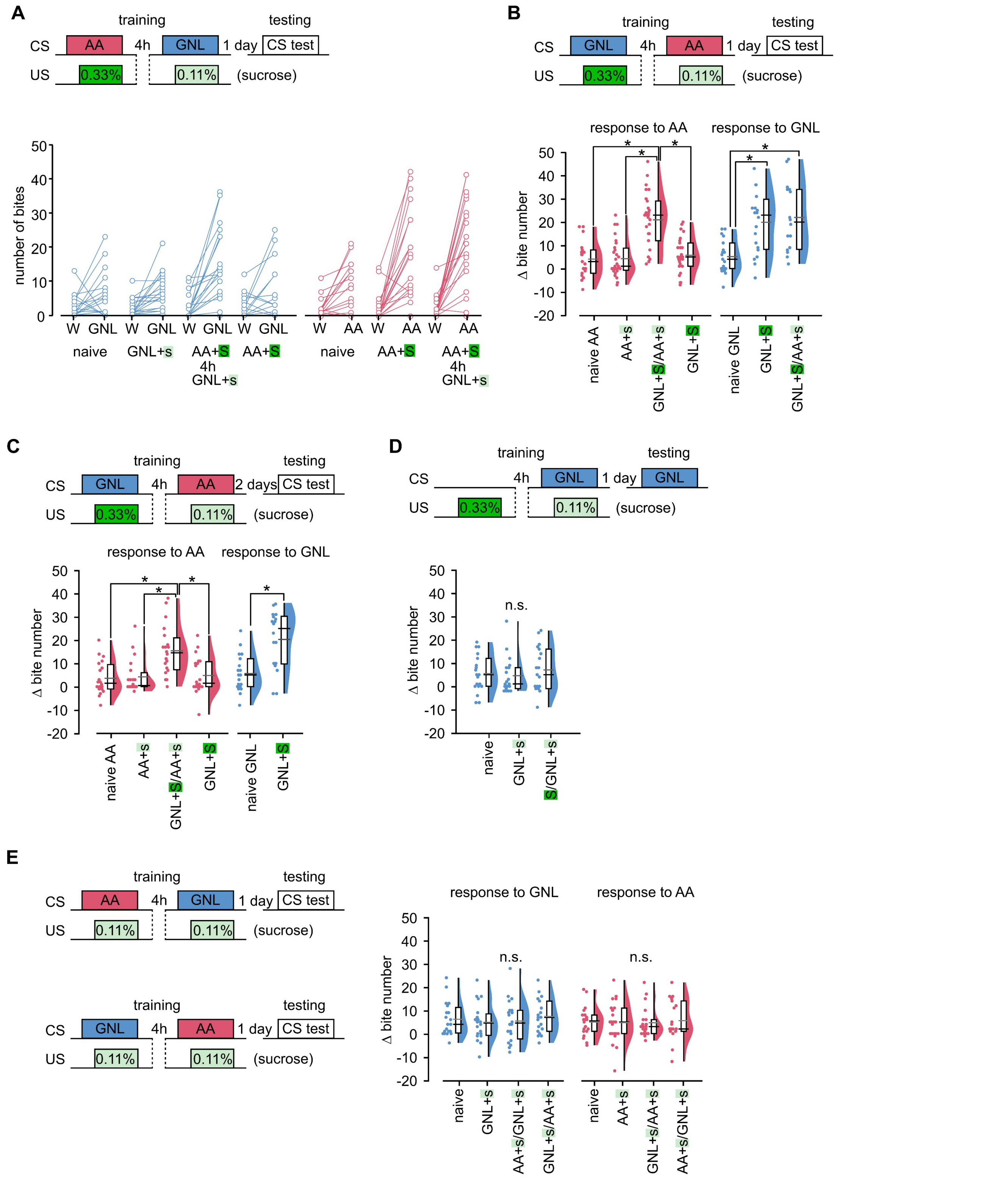

Fig. S2. Dependency of weak-training LTM formation on acquisition of a prior strong memory.

(**A**) Presentation of data showing the bite numbers in response to water and the CS used to calculate the Δ bite number in Figure 1D. Schematic of timeline of experiment (top).

(**B**) Switching the CSs used for strong and weak training also induces a LTM for weak training. Schematic timeline of experiment (top). Strong training using GNL as the CS 4 h before weak training using AA as the CS causes a significant learned response to the CS AA compared to naïve, weak training only or strong training only (naïve; n = 19, weak training only; n = 28, strong 4 h before weak; n = 27, strong training only; n = 27, Kruskal-Wallis test,

$p < 0.001$, H = 33.3; Dunn's test: strong 4 h before weak vs naïve $p < 0.001$, weak only $p < 0.001$, and strong only $p < 0.001$, all other conditions $p > 0.05$). Strong followed by weak training also results in a significant increase in response to the CS GNL compared to naïve, but not to strong training only (naïve; n = 19, strong only; n = 20, strong 4 h before weak; n = 16; Kruskal-Wallis test, $p < 0.001$, H = 16.97; Dunn's test: strong 4 h before weak vs naïve, $p < 0.001$, strong only vs naïve $p < 0.001$, strong 4 h before weak vs strong only $p > 0.05$).

(**C**) Strong followed by weak training induces a memory for the weak training which persists for at least 48 h. Schematic timeline of experiment (top). Strong training using GNL as the CS 4 h before weak training using AA as the CS causes a significant learned response to the CS AA when tested 48 h later compared to naïve, weak training only or strong training only (naïve; n = 20, weak training only; n = 20, strong 4 h before weak; n = 20, strong training only; n = 20, Kruskal-Wallis test, $p < 0.001$, H = 19.45; Dunn's test: strong 4 h before weak vs naïve $p < 0.001$, weak only $p < 0.001$, and strong only $p < 0.01$, all other conditions $p > 0.05$). Strong training results in a significant increase in response to the CS GNL when tested 48 h later compared to naïve (naïve; n = 19, strong only; n = 18; Mann Whitney test, $p < 0.001$, U = 60.5).

(**D**) Application of the strong US alone is not sufficient to enable weak-training LTM formation. Schematic timeline of experiment (top). Presentation of the US used in the strong training alone (0.33% sucrose) 4 h before weak training does not lead to a significant increase in the response to the CS GNL compared to naïve or weak training only (n = 19 for all conditions, Kruskal-Wallis test, $p > 0.05$, H = 0.75).

(**E**) Prior weak training is not sufficient to enable subsequent weak-training LTM formation. Schematic timeline of experiment (left). Using AA as the CS and 0.11% sucrose as the US as the first weak training 4 h before the second weak training using GNL as the CS and 0.11% sucrose as the US did not cause a significant learned response to the CS GNL when tested 24 h later compared to naïve, weak training only (GNL+0.11% sucrose) or reversing the order of weak training (GNL+0.11% sucrose first training, AA+0.11% sucrose second training) (naïve; n = 20, weak training only; n = 20, AA+0.11% sucrose 4 h before GNL+0.11% sucrose; n = 20, GNL+0.11% sucrose 4 h before AA+0.11% sucrose; n = 19, Kruskal-Wallis test, $p > 0.05$, H = 0.77). Using GNL as the CS and 0.11% sucrose as the US as the first weak training 4 h before the second weak training using AA as the CS and 0.11% sucrose as the US did not cause a significant learned response to the CS AA when tested 24 h later compared to naïve, weak training only (AA+0.11% sucrose) or reversing the order of weak training (AA+0.11% sucrose first training, GNL+0.11% sucrose second training) (naïve; n = 20, weak training only; n = 22, GNL+0.11% sucrose 4 h before AA+0.11% sucrose; n = 20, AA+0.11% sucrose 4 h before GNL+0.11% sucrose; n = 19, Kruskal-Wallis test, $p > 0.05$, H = 0.36).

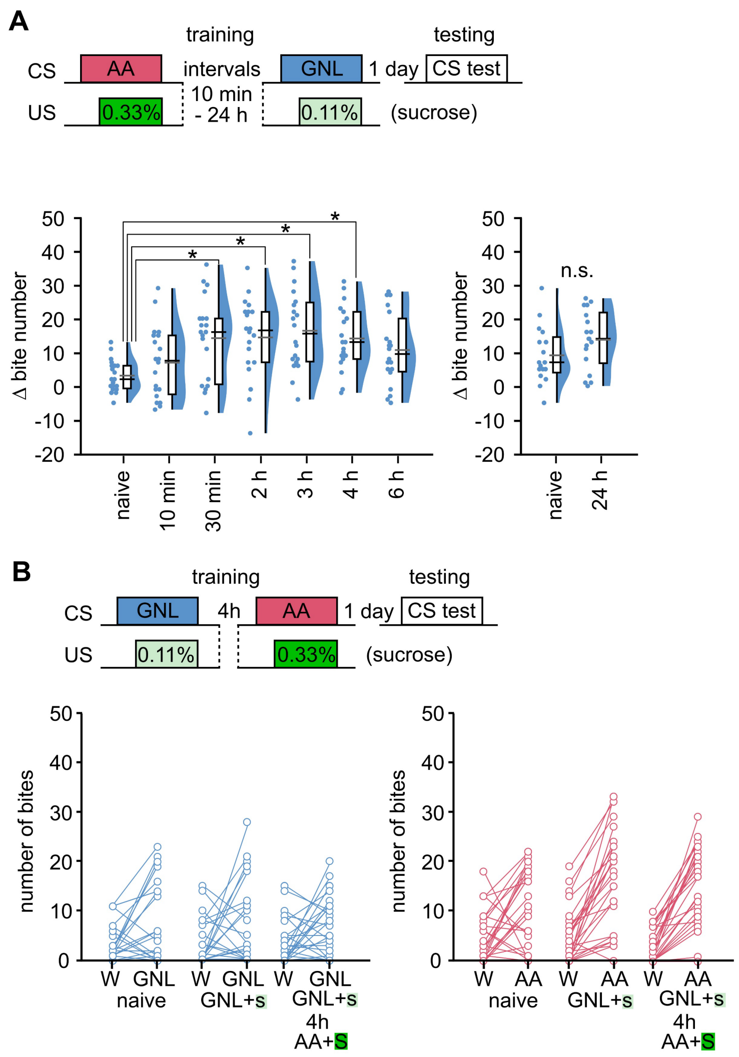

Fig. S3. Characterization of time course over which strong training enhances weak training memory acquisition.

(**A**) Varying interval between strong and weak training influences the outcome of weak-training learning. Schematic of experimental timeline (top). Animals received the weak training at 10 min (n = 20), 30 min (n = 20), 2 h (n = 20), 3 h (n = 20), 4 h (n = 19) or 6 h (n = 20) after the strong training. There was a significantly higher feeding response to the CS GNL when weak training was presented 30 min, 2 h, 3 h and 4 h after the strong training, but not at 10 min or 6 h compared to naïve (n = 20) controls (One-way ANOVA, $p < 0.001$ ($F(6,138) = 4.3$), Tukey's test, naïve versus 30 min $p < 0.05$, versus 2 h $p < 0.05$, versus 3 h $p < 0.01$, versus 4 h $p < 0.05$; all other conditions $p > 0.05$). A second group of animals received the weak training 24 h after the strong training and were tested a further 24 h (n = 17) later showing no significant difference compared to naïve (n = 18) controls (two-tailed t-test $p > 0.05$, t = 1.65).

(**B**) Presentation of data showing the bite numbers in response to water and the CS used to calculate the Δ bite number in Figure 1E. Schematic of timeline of experiment (top).

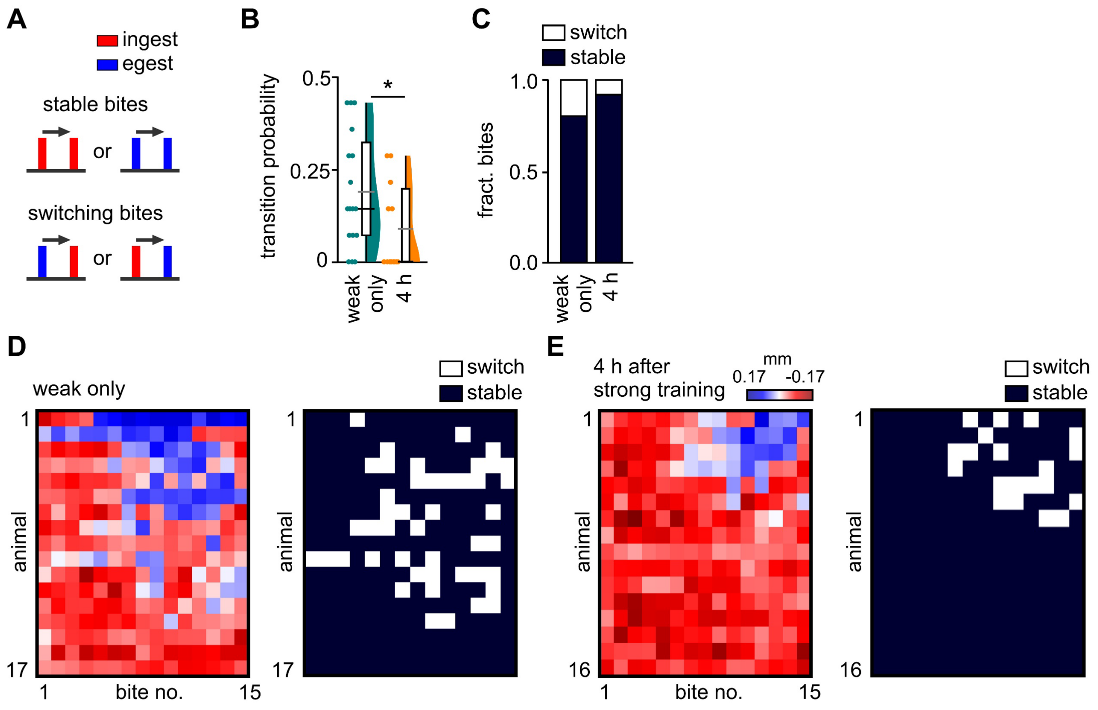

Fig. S4. Prior strong learning stabilizes the perception of the CS+US during subsequent weak training.

(**A**) Schematic of bite classification. Bites were classified as ingestion or egestion as in Figure 2. If an ingestion bite was followed by an ingestion bite or an egestion bite was followed by an egestion bite it was classified as a stable pair of bites. If an egestion bite was followed by an ingestion bite or an ingestion bite was followed by an egestion bite it was classified as a switch.

(**B**) Bites had a higher transition probability in animals receiving weak training only compared to animals which received strong training 4 h earlier (Mann Whitney test, $p < 0.05$, U = 82.5).

(**C**) Comparison of the fraction of stable versus switch bites showed a significant difference between conditions (Fisher's exact test $p < 0.01$).

(**D** and **E**) Heat plots of bites during weak training as in Figure 2B with corresponding plots of stable bite pairs and switching bite pairs for animals receiving weak training only or weak training 4 h after strong training.

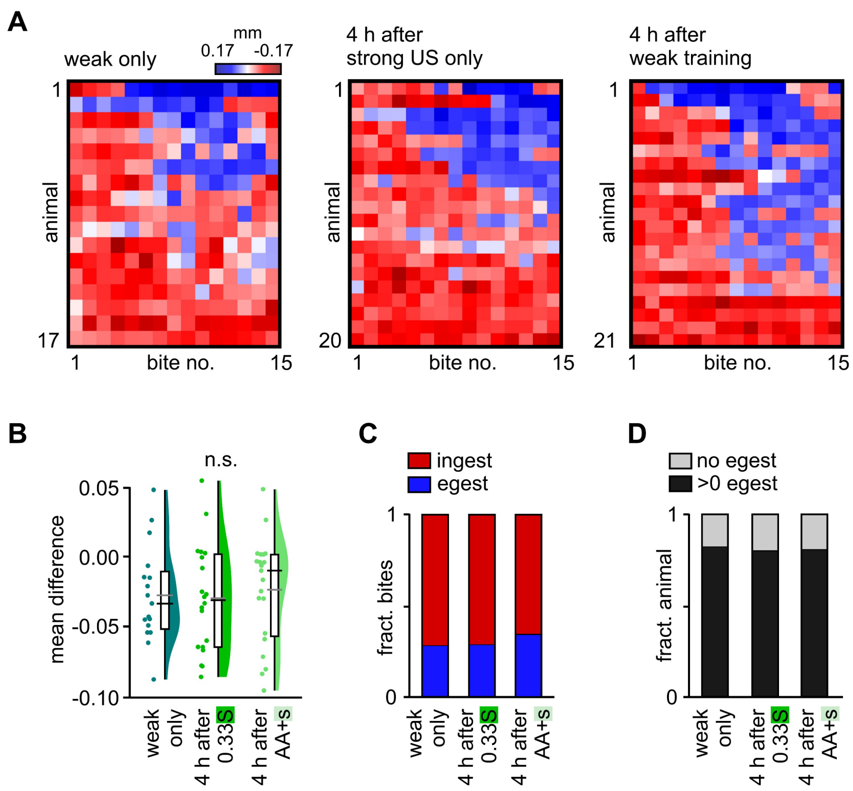

Fig. S5. The shift in perception of weak training is dependent on prior acquisition of a strong memory.

(**A**) Heat-plots of radula movements recorded during the first 15 bites in response to the CS+US (GNL+0.11% sucrose) in animals receiving the weak training only compared to animals which received the strong training US (0.33% sucrose) 4 h earlier or weak training (AA+0.11% sucrose) 4 h earlier. Red-white-blue lookup table represents movements of distal tip of radula. Positive values (blue) are egestion cycles; negative values (red) are ingestion cycles.

(**B**) Statistical summary of (A) shows no significant change in the mean difference in radula movements between groups (One-way ANOVA, $p > 0.05$ ($F(2,57) = 0.17$)).

(**C**) Plot of fraction of ingestion and egestion bites in response to the weak training showing no significant difference between groups (Fisher's exact test, $p > 0.05$).

(**D**) Plot of fraction of animals performing no egestion bites versus at least one egestion bite in response to weak training showing no significant difference between groups (Fisher's exact test $p > 0.05$).

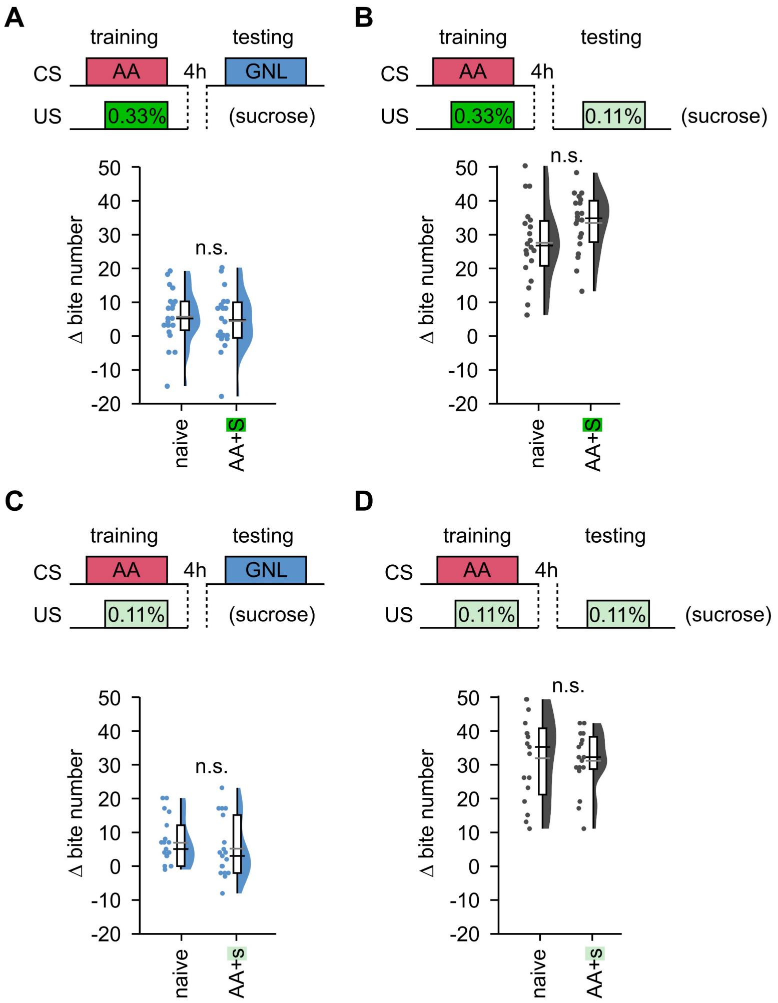

Fig. S6. Past strong or weak training does not alter the general responsiveness to either the CS or US used for weak training.

(**A** and **B**) Strong training alone does not alter the Δ bite number in response to the weak training CS (GNL) (naïve; n = 20, strong trained; n = 20, two-tailed t-test $p > 0.05$, t = 0.39) or US (0.11% sucrose) (naïve; n = 20, strong trained; n = 20, two-tailed t-test $p > 0.05$, t = 1.88) when tested 4 h after strong training.

(**C** and **D**) Weak training alone does not alter the Δ bite number in response to the weak training CS (GNL) (naïve; n = 19, strong trained; n = 19, Mann Whitney test, $p > 0.05$, U = 140.5) or US (0.11% sucrose) (naïve; n = 17, strong trained; n = 17, two-tailed t-test $p > 0.05$, t = 0.19) when tested 4 h after weak training.

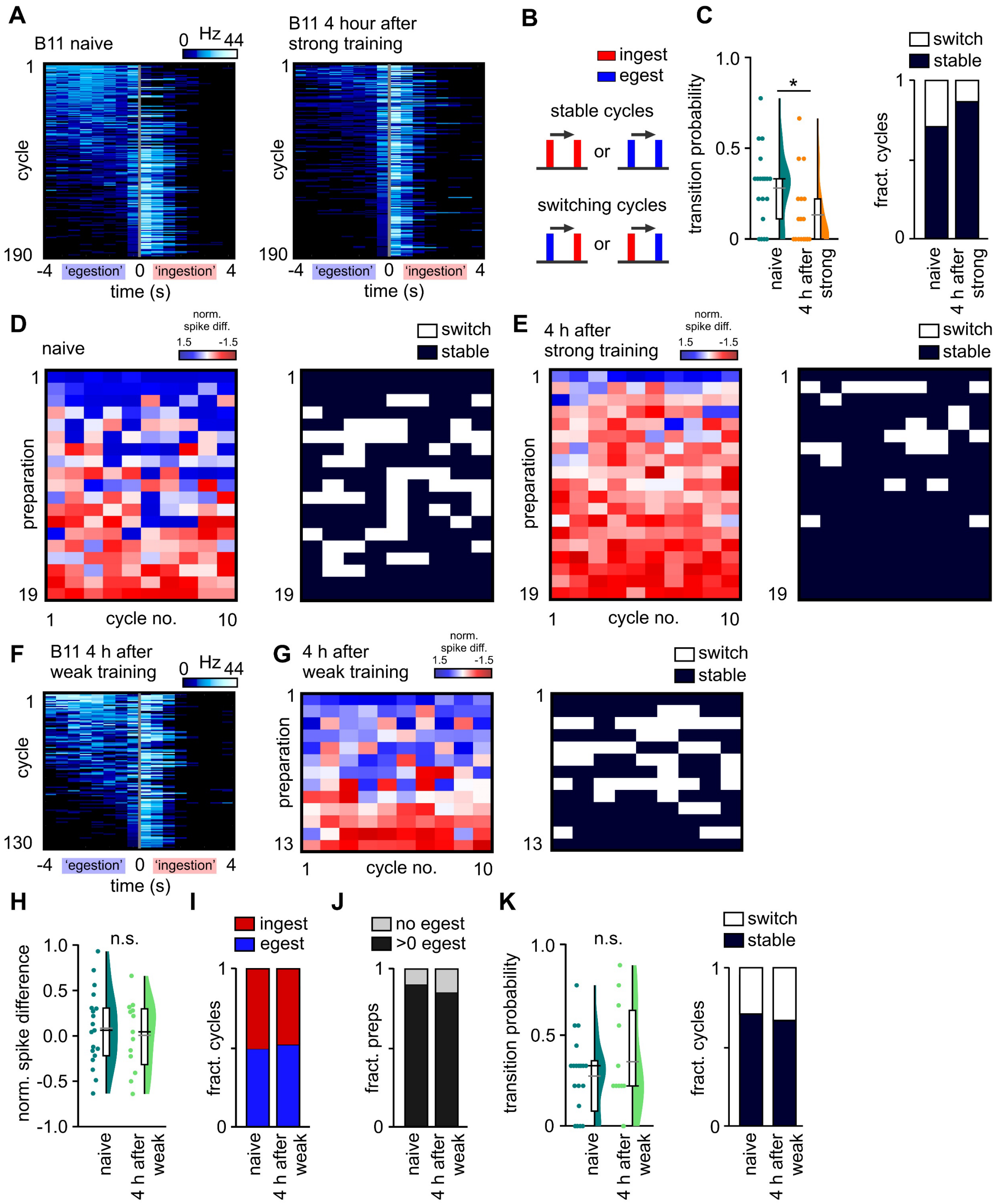

Fig. S7. Neural correlates of network state: prior strong training leads to a stabilization of the network state that favours ingestion behaviour whereas prior weak training does not.

(**A**) Heatplots of B11 activity in multiple trials during *in vitro* cycles from preparations from naive animals (left) and from animals which had received strong training 4 h earlier (right). Gray lines represent onset of retraction phase. Data are ordered from high to low activity for the period before retraction starts. Predominant activity on left side of line is associated with egestion responses and activity on the right corresponds to ingestion events.

(**B**) Schematic of cycle classification. Cycles were classified as ingestion or egestion as in Figure 2. If an ingestion cycle was followed by an ingestion cycle or an egestion cycle was followed by an egestion cycle it was classified as a stable pair of cycles. If an egestion cycle was followed by an ingestion cycle or an ingestion cycle was followed by an egestion cycle it was classified as a switch.

(**C**) Summary plots. (left) Cycles had a higher transition probability in preparations from naïve compared to preparation from animals which received strong training 4 h earlier (Mann Whitney test, $p < 0.05$, U = 102). (right) Comparison of the fraction of stable versus switch cycles showed a significant difference between conditions (Fisher's exact test $p < 0.05$).

(**D** and **E**) Heat plots of spontaneous cycles as in Figure 2G (left) with corresponding plots of stable cycle pairs and switching cycle pairs for naïve and trained preparations (right).

(**F**) Heatplots of B11 activity in multiple trials during *in vitro* cycles from preparations from animals which received weak training (AA+0.11% sucrose) 4 h earlier. Gray lines represent onset of retraction phase. Predominant activity on left side of line is associated with egestion responses and activity on the right corresponds to ingestion events.

(**G**) Heat plots of spontaneous cycles (left) with corresponding plots of stable cycle pairs and switching cycle pairs (right) for weak trained preparations.

(**H**) Statistical summary of (G) shows no significant change in B11 spike difference in preparations from naïve animals versus those from animals that received weak training 4 h earlier (two-tailed t-test $p > 0.05$, t = 0.5).

(**I**) Plot of fraction of ingestion and egestion cycles showing no significant difference between naïve preparations and those from animals that received weak training 4 h earlier (Fisher's exact test $p > 0.05$).

(**J**) Plot of fraction of preparations producing no egestion cycles versus at least one egestion cycle showing no significant difference in naïve preparations versus those from animals that received weak training 4 h earlier (Fisher's exact test $p > 0.05$).

(**K**) (left) Cycles did not have a different transition probability in preparations from naïve animals compared to animals that received weak training 4 h earlier (two-tailed t-test, $p > 0.05$, t = 0.52). (right) Comparison of the fraction of stable versus switch cycles showed no significant difference between conditions (Fisher's exact test $p > 0.05$).

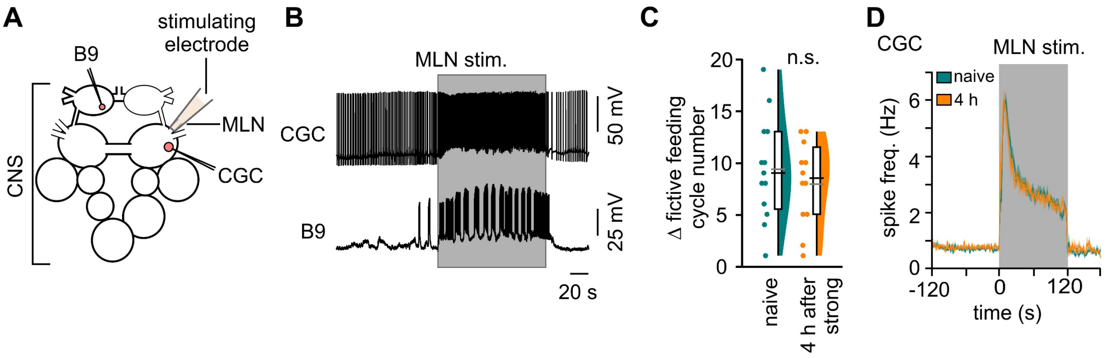

Fig. S8. Prior strong training does not alter the responsiveness of the feeding circuitry.

(**A**) Cartoon of *in vitro* preparation used to test for fictive feeding and modulatory interneuron response properties to lip nerve stimulation.

(**B**) Example traces of the serotonergic modulatory interneuron CGC and a feeding motoneuron B9 in response to stimulation of the medial lip nerve (MLN), which contain chemosensory fibres from the lips. Nerve stimulation causes an acceleration of the tonic firing rate of the CGC, and a bout of fictive feeding cycles recorded on B9.

(**C**) Summary analysis plot. There was no significant difference in the number of fictive feeding cycles triggered by medial lip nerve stimulation between naïve preparations (n = 13) and preparations from animals which had received strong training 4 h earlier (n = 12, two-tailed t-test, $p > 0.05$, t = 0.8). Δ fictive feeding cycle number was acquired by subtracting the number of spontaneous cycles 2 min before medial lip nerve stimulation from the number of cycles produced during the 2 min medial lip nerve stimulation.

(**D**) Plot of CGC spike frequency. There was no significant difference in tonic firing rates before the stimulus (naïve; n = 15, 4 h; n = 12, two-tailed t-test, $p > 0.05$, t = 0.31) or a difference between spike frequency during the stimulus (two-tailed t-test, $p > 0.05$, t = 0.47) between conditions. Line shows mean, shading shows ±SEM.

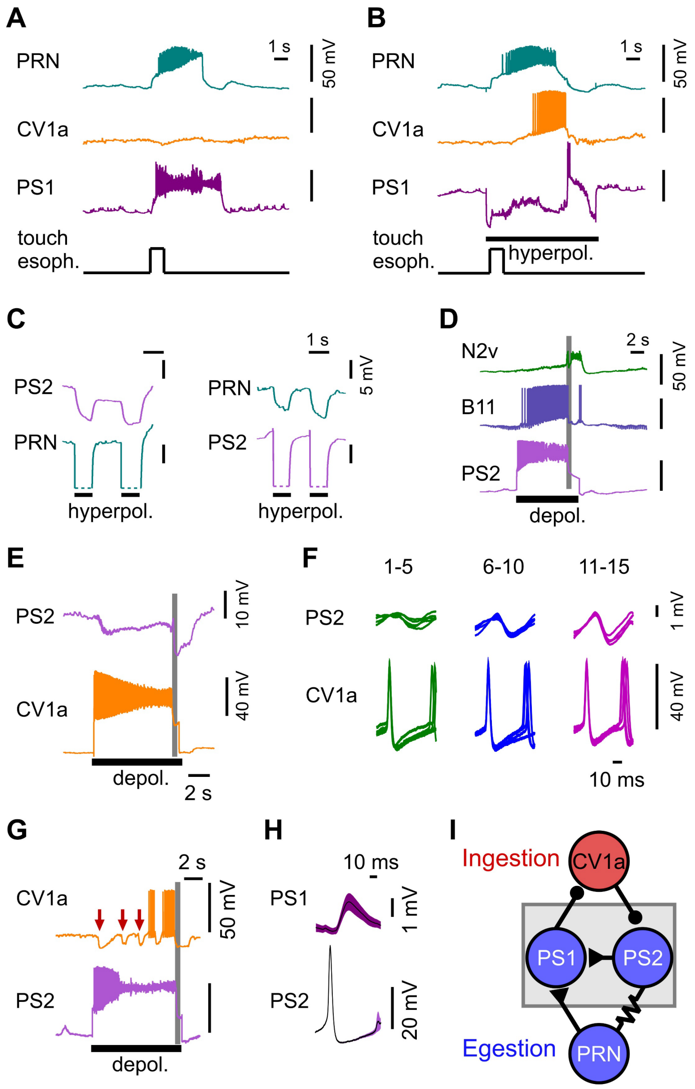

Fig. S9. The perceptual control circuit mediates competitive interactions via a mutual inhibition motif.

(**A**) PS1 activation during sensory-driven egestion. Tactile stimulation of the esophagus can be used to drive robust egestion cycles *in vitro*. Example trace of PS1 co-recorded with the ingestion command-like interneuron CV1a and the egestion command-like interneuron PRN. Both PS1 and PRN are strongly active during the sensory-driven cycle whereas CV1a shows no spiking activity.

(**B**) Artificial hyperpolarization of PS1 during a sensory-driven cycle results in CV1a being recruited into the cycle, demonstrating that it is the key source of inhibition during egestion cycles. CV1a spike frequency was significantly greater during touch to esophagus driven cycles where PS1 was artificially hyperpolarized (n = 5, Wilcoxon test, $p < 0.05$, W = 15).

(**C**) Electrotonic coupling of PRN and PS2. Application of hyperpolarizing square current pulses to PRN or PS2 causes similar but reduced responses in the other neuron.

(**D**) Artificial activation of PS2 drives an egestion fictive feeding cycle as seen by B11 activity occurring predominantly in the protraction phase. Gray line represents the onset of the retraction phase.

(**E**) During CV1a-driven ingestion, PS2 is strongly inhibited.

(**F**) CV1a spikes cause facilitating inhibitory postsynaptic potentials on PS2.

(**G**) Artificial activation of PS2 causes large waves of inhibition on CV1a which do not arise directly from PS2 activity.

(**H**) PS2 caused 1:1 EPSPs on PS1.

(**I**) Schematic summarizing organization and wiring of control network that determines ingestion-egestion balance. Box indicates the components of the switch circuit that control competitive interactions.

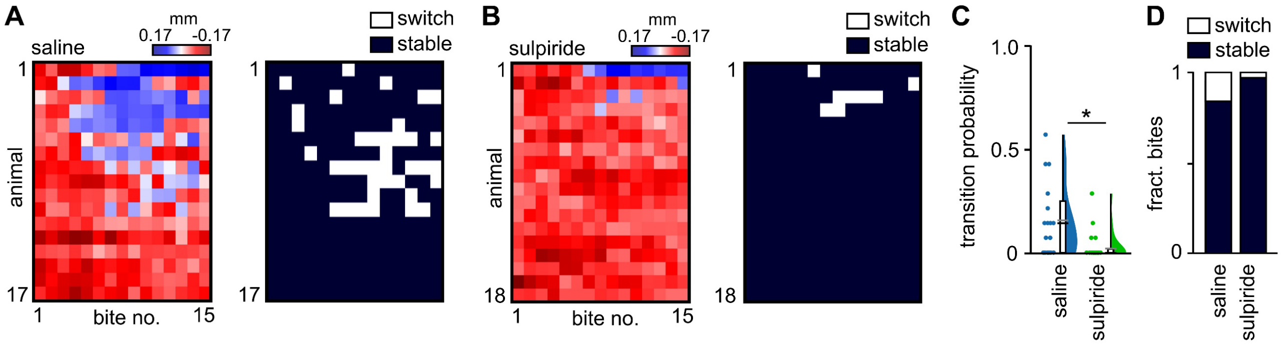

Fig. S10. Pharmacologically altering the network state stabilizes the perception of weak training.

(**A** and **B**) Heat plots of bites in response to the CS+US during weak training as in Figure 5E with corresponding plots of stable bite pairs and switching bite pairs for animals receiving weak training after saline injection or sulpiride injection.

(**C**) Bites had a higher transition probability in animals injected with saline compared to animals injected with sulpiride (Mann Whitney test, $p < 0.01$, U = 79.5).

(**D**) Comparison of the fraction of stable versus switch bites showed a significant difference between conditions (Fisher's exact test $p < 0.01$).

Movie S1. Conditioned feeding response in naïve and trained animals.

Video showing memory expression readout after appetitive conditioning. Underside view of a naïve (left) and a trained (1 day after training, right) animal in response to CS (AA) application. The naïve animal shows no feeding behaviours whereas the trained animal produces a bout of rhythmical feeding activity (opening/closing of the mouth and protraction/retraction of the radula) demonstrating that the animal has acquired a LTM. Video x4 speed.
